# Dichloromethane extracts of propolis protect cell from oxygen-glucose deprivation-induced oxidative stress via reducing apoptosis

**DOI:** 10.3402/fnr.v60.30081

**Published:** 2016-06-20

**Authors:** Li-Ping Sun, Xiang Xu, Hau-Hsuan Hwang, Xin Wang, Kang-Yi Su, Yi-Lin S. Chen

**Affiliations:** 1 Institute of Apicultural Research, Chinese Academy of Agricultural Sciences, Beijing, China; 2 Department of Life Sciences, National Chung Hsing University, Taichung, Taiwan; 3 Department of Clinical Laboratory Sciences and Medical Biotechnology, College of Medicine, National Taiwan University, Taipei, Taiwan; 4 Department of Biotechnology and Animal Science, National Ilan University, Ilan, Taiwan

**Keywords:** propolis, OGD, ischemia-reperfusion injury, anti-apoptosis, oxidative stress

## Abstract

**Background:**

Bee propolis, a mixture of the secretion from bee tongue gland and wax gland, was collected from the tree bud and barked by bees. The components were rich in terpenes, phenolics, and flavonoids, and had anti-cancer, anti-bacterial, anti-inflammatory, hepatoprotective, and neuroprotection abilities. However, the potential anti-oxidative stress of propolis was not well documented. This study aimed to study the protective effect of propolis on high-incident nonfatal diseases, such as stroke and cerebral infarction caused by ischemia.

**Objective:**

Oxidative stress caused by acute stroke results in inflammation and injury followed by cell damage and apoptosis. Clarification of the anti-oxidative stress effect of propolis may contribute to stroke prevention and damage reduction.

**Design:**

Propolis was separated and purified into 70% ethanol and dichloromethane extracts systematically. The fraction three (Fr.3) of dichloromethane was further separated into pinocembrin, pinobanksin, pinobanksin-3-acetate, chrysin, and galangin by chromatography. Compounds extracted from propolis were tested for cell-protection effects in an oxygen-glucose deprivation (OGD) N2a cell model. MTT assay, oxidative stress markers measurement, flow cytometry, and QPCR were used to evaluate cell viability and apoptosis.

**Results:**

All compounds, especially pinocembrin and galangin, enhanced cell viability in OGD-treated N2a cells. In addition, anti-oxidative enzymes were elevated and cellular Ca^2+^ was reduced. They also had extreme anti-apoptosis effects by up-regulating the expression of Bcl-2 mRNA and down-regulating caspase-3 and Bax expression. Taken together, propolis had anti-oxidative effects on stress and protected cells from damage.

**Conclusion:**

The anti-oxidative effect of propolis can be applied to daily food supplements and may benefit stroke patients.

Stroke, especially ischemic stroke, is the first of the three leading causes of death worldwide. Cerebral reperfusion is the most popular therapeutic strategy for patients with acute stroke. However, cerebral ischemia/reperfusion (I/R) also causes neural cell damage known as reperfusion injury (1). The pathogenesis of injury due to I/R is very complex and the underlying mechanisms widely involve cell apoptosis, inflammation, as well as oxidative stress (2). More and more evidence indicates that various cytokines and stress-induced factors, such as 17β-estradiol that can inhibit apoptosis of cardiomyocyte or macrophage inhibitory cytoline-1 that can protect dopaminergic neurons from damage (3, 4). On the contrary, reactive oxygen species (ROS) is also significant in the process of injury due to its association with the brain's metabolic signaling and high oxygen consummation (5). Furthermore, oxidative stress related to oxygen-derivate free radicals may result in an imbalance in the hemostasis between pro-oxidants and antioxidants that can cause cell damage, autophagy, apoptosis, as well as necrosis during stroke (6). Superoxide dismutase (SOD), catalase (CAT), and glutathione peroxidase (GPx) act by scavenging the superoxide anion and H_2_O_2_ to prevent ROS-induced damage (7). In animal experiments, the clearance of free radicals by scavenger enzymes can efficiently attenuate the apoptosis-associated DNA fragmentation damage in the cerebral ischemia mice model (8). Although to efficiently eliminate the I/R-induced damage is still challenging and difficult, two specific aims are focused on solving these problems. One is to understand the molecular mechanism for novel drug development and the other is to identify the supplemental food for adjuvant therapy in the clinical practice (9).

Propolis is a natural product with complex mixed compounds collected and produced by the honeybees (*Apis mellifera*) to seal cracks, smooth walls, and block moisture. Naturally, bees use propolis as a general sealer, draft excluder, antibiotic, and embalming substance for the carcasses of hive invaders. It is temperature-stable in the hive all year around (10). The common colors of propolis were yellow, green, brown, and black, but vary greatly according to the source and season in which it is collected (11). Propolis contains ~80% plant resins and waxes and ~20% substances with biological applications such as aromatic oils, pollens, phenolic acid, flavonoids, and other organic substances (12, 13). With the improvement and development of purification techniques, most compounds can be further identified and quantified by layer chromatography, gas chromatography (GC), high-performance liquid chromatography (HPLC), mass spectrometry (MS), nuclear magnetic resonance (NMR), and GC-MS (14, 15). Despite the popularity of propolis over time, its many biological properties provided not only supplements but also a dietary component for complementary or alternative medicine. To date, more and more evidence shows the therapeutic effects of propolis on the treatment of colds, wounds, diabetes, and cardiovascular diseases (16–18). These applications are based on anti-tumor (19), antioxidant (20), antibacterial (21), antiviral (22), antifungal (23), and anti-inflammatory properties (24). Our previous studies also exhibited that propolis can protect the liver from TGF-β stimulated fibrosis damage by down-regulating JNK and Smad2/3 signaling (25). However, the effect of propolis on oxidative stress had not been well documented. In this study, we examined the neuroprotective effect of propolis in an oxygen-glucose deprived (OGD) cell model in N2a cells to mimic cerebral I/R injury. The results indicated that the compounds extracted from raw propolis can be a potential antioxidant agent by attenuating cell apoptosis. This highlights the further application of propolis in therapeutic food supplements.

## Material and methods

### Origin of propolis

Chinese propolis (CP) was collected in Shandong. Samples were maintained at −20°C before processing.

### Extraction and purification of CP

The procedure of extraction and purification was based on previous studies (25–27). Briefly, the CP samples (50 g) were treated and homogenized by stirring at 4°C. The residue was extracted three times with 40, 70, and 95% ethanol followed by sonication for 3 h. The filtered ethanol extract was evaporated under reduced pressure to obtain a brown powder, which was stored at −20°C until further purification.

### Analytical conditions for HPLC

The chemical compositions of chrysin and CAPE in the CP samples were analyzed by using a reverse-phase preparation of HPLC. The separation conditions were as follows: column, Luna Phenomenex C18 (250 mm×4.6 mm; USA); mobile phase, methanol:water (55:45); flow rate, 1.0 mL/min; detection, UV 280 nm; injection volume, 20 µL.

### LC-nana ESI-Q-TOF MS analysis

The separated compounds from fraction three (Fr.3) by HPLC were analyzed on-line under the positive survey scan mode on a nano-ESI Q-TOF (Micromass, UK) instrument with the following conditions: 4,000 V for ESI voltage, 320°C and 35 psi for atomization, 6 L/min for drying gas flow, and 350°C and 9 L/min for sheath flow. The scan range was from m/z 100–1,000. The raw data was processed into a text file format of PKL with MassLynx 4.0 (subtract 30%, smooth 3/2 Savitzky Golay and center three channels 80% centroid).

### Cell culture

N2a cells were obtained from the China Infrastructure of Cell Line Resources, Beijing. The cells were propagated in DMEM medium (Thermo) supplemented with 10% heat-inactivated fetal calf serum (GIBICO), and 1% penicillin-streptomycin at 37°C in a 5% CO_2_ incubator.

### Cell viability assays

The cell viability assay was performed by the CCK-8 assay based on the conversion of CCK-8 into formazan crystals by living cells, which determined mitochondrial activity. Cells were cultured in a 96-well plate with 5,000/well confluence. Twenty-four hours after treatment, 10 µL CCK-8 reagents were added for 1 h incubation, followed by ELISA reader detection at 450 nm. The data was represented by three independent experiments. The cell viability of CCK-8 was calculated using the following equation A1 for each experimental group absorbance values; A2 for the absorbance values of the control group; A0 absorbance values for the blank group):

$$\text{Cell viability}=\left(\frac{\text{A}1-\text{A}0}{\text{A}2-\text{A}0}\right)\times 100\%$$

### Giemsa stain

Giemsa's solution is a mixture of methylene blue, eosin, and Azure B. The stain is usually prepared from commercially available Giemsa powder. A thin film of the specimen on a microscope slide is fixed in pure methanol for 2–3 min, by immersing it or putting a few drops of methanol on the slide. The slide is immersed in a freshly prepared 10% Giemsa stain solution for 15–30 min, then flushed with tap water and left to dry.

### Oxidative enzyme and biomarker measurement

For superoxide dismutase measurement, SOD Assay Kit (Cat.19160) (Sigma-Aldrich, MO, USA) was utilized according to manufacturer's instructions. Briefly, a 20 µL sample solution was mixed with 200 µL of WST-1 (2-(4-iodophenyl)-3-(4-nitrophenyl)-5-(2,4-disulfophenyl)-2H-tetr azolium, monosodium salt) and 20 µL of xanthine oxidase in a 96-well plate, followed by incubation at 37°C for 20 min, and the absorbance was measured at 450 nm. GPx activity was determined using the GPx activity assay kit (BioVision Inc., Milpitas, CA, USA) according to the manufacturer's protocol, and the absorbance was measured at 340 nm.

### Flow cytometry analysis

The N2a cell apoptosis was assessed using the Annexin V-FITC Apoptosis Detection Kit (Sigma-Aldrich, MO, USA) according to manufacturer's instructions. After treatment, N2a cells were harvested, washed with PBS, and resuspended in a binding buffer containing FITC-conjugated Annexin V and PI. After 30-min incubation in the dark, cells were determined using flow cytometry (Becton Dickinson LSRII). The percentages of the cells residing in the lower right (apoptotic cells) and upper right (necrotic cells) regions of the Annexin V-FITC scatter plots were calculated for further analysis.

### Oxygen glucose deprivation model

The N2a cells were randomly divided into the normal group, the model group, and the positive control group. In addition to the normal group, the model group and the positive control group were treated. The positive control group advance to 10 µg/mL working solution was incubated for 24 h, the model group and the appositive control groups of cells were equilibrated with the advance in hypoxic devices replace the original sugar-free Earle liquid medium into the hypoxic devices, hypoxia for 1 h, 2 h, 4 h, and 6 h, respectively, and then washed three times in PBS with DMEM high-sugar replacement sugarless Earle's culture fluid, and the cells were then returned to the incubator with 5% CO_2_ and incubated for 24 h.

### Thin-layer chromatography assay

Samples (2–5 µL) were spotted onto a silica gel TLC plate 6–7 mm apart and developed with chloroform/methanol/formic acid (9/0.6/0.5 by volume), followed by drying.

### Statistical analysis

All experiments are performed in triplicate and the values are given as means±S. E. Data analysis involved one-way ANOVA with subsequent Scheffé test. *p* value <0.05 is considered statistically significant.

## Results

### Alcohol-soluble extracts of propolis attenuate OGD induced cell damage

**Fig. 1:**
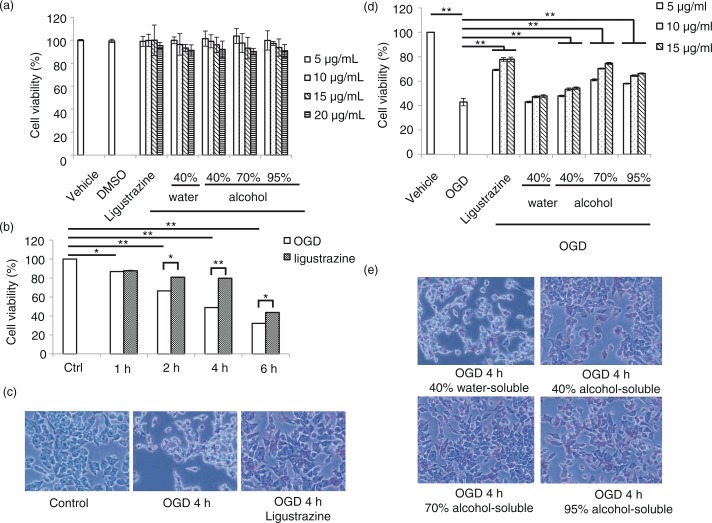
Extracts of propolis attenuate oxygen-induced-deprivation (OGD)-induced cell damage. a) Cytotoxicity test of water- or alcohol-soluble extracts from propolis in N2a cell. Ligustrazine was a control for cell protection. b) Optimization of the effect of OGD on cell damage in different time courses. c) Illustration of cell death 4 h after OGD treatment. Ligustrazine can protect cell from damage. d) The effect of water- or alcohol-soluble extracts from propolis on OGD-induced cell damage. Ligustrazine was a positive control for cell protection. e) Image of cell phenomena for water- or alcohol-soluble extracts from propolis in cell-protection effects. *n*=3 for each group; *p<0.05; **p<0.01.

In order to evaluate the role of propolis in cell damage, the model of OGD-induced cell damage on an N2a (Neuro-2a) neuroblastoma cell line was utilized. First, the cytotoxicity of propolis was tested. N2a cells were treated with different dosages of water- or alcohol-soluble fractions from propolis extracts followed by MTT cell viability assay (Fig. 1a). The result indicated that the extracts from propolis exhibited low cell cytotoxicity. The cell viability was more than 80% at the concentration as high as 20 µg/ml. To optimize the effect of OGD on cell damage, different time points after OGD treatment was evaluated (Fig. 1b). After OGD treatment, the cell viability of N2a cells were decreased in a time-dependent manner while ligustrazine was used as a positive control for cell protection. Ligustrazine can attenuate cell damage and significantly enrich more viable cells 4 h after OGD treatment (Fig. 1c). Hence, we further tested the effect of water- or alcohol-soluble fractions from propolis on cell protection to eliminate the OGD-induced damage (Fig. 1d). We found that the alcohol-soluble fraction, but not the water-soluble fraction can protect cell from damage in a dosage-dependent manner. N2a cells treated with 70% alcohol-soluble extracts from propolis had relative abundant viable cells 4 h after OGD challenge compared with the 40 or 90% alcohol-soluble extracts (Fig. 1e).

### Gradient extracts from 70% alcohol-soluble fraction can protect cell from OGD-induced damage

**Fig. 2:**
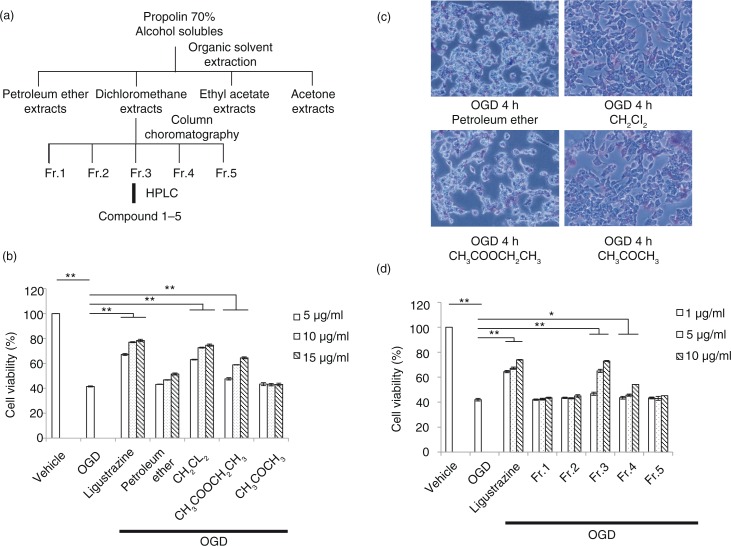
The protection effect of serial extracts from propolis on OGD-induced cell death. a) The procedure of extraction by organic solvent and column chromatography. b) Cell-protection effects by extracts with different doses, including petroleum ether, dichloromethane, ethyl acetate, and acetone from propolis after organic solvent extraction. c) Cell morphology for protection effects by propolis extracts in N2a cell 4 h after OGD treatment. d) Cell protection assay in an OGD-treated N2a cell of fraction one to five from dichloromethane extracts of propolis by column chromatography. *n*=3 for each group; *p<0.05; **p<0.01.

Based on the above results, we further purified the 70% alcohol-soluble fraction of propolis to identify pure extracts for cell protection. The procedure of extraction was followed by standard organic solvent fractional extraction (Fig. 2a). The extracts from dichloromethane were further separated by column chromatography to identify a fraction one to five (Fr.1–5). Finally, the pure compound was concentrated by HPLC. First, organic extracts including petroleum ether, dichloromethane, ethyl acetate, and acetone were tested for cell protection against OGD-induced damage. Among all extracts, the dichloromethane extract exhibited the most significant effect to attenuate OGD-induced cell death in a dosage-dependent manner (Fig. 2b). Therefore, it was assessed for further purification. After the OGD challenge, cells with extracts of the propolis treatment, especially the dichloromethane extract, exhibited more viability (Fig. 2c). Furthermore, among all five fractions separated by HPLC, fraction three (Fr.3) showed the most efficacy, in a dosage-dependent manner, for cell protection after the OGD challenge (Fig. 2d). These results indicated that the specific extract from propolis has the potential to protect cells from environmental oxidative stress and toxics.

### Compounds extracted from propolis can significantly reduce OGD-induced damage

**Fig. 3:**
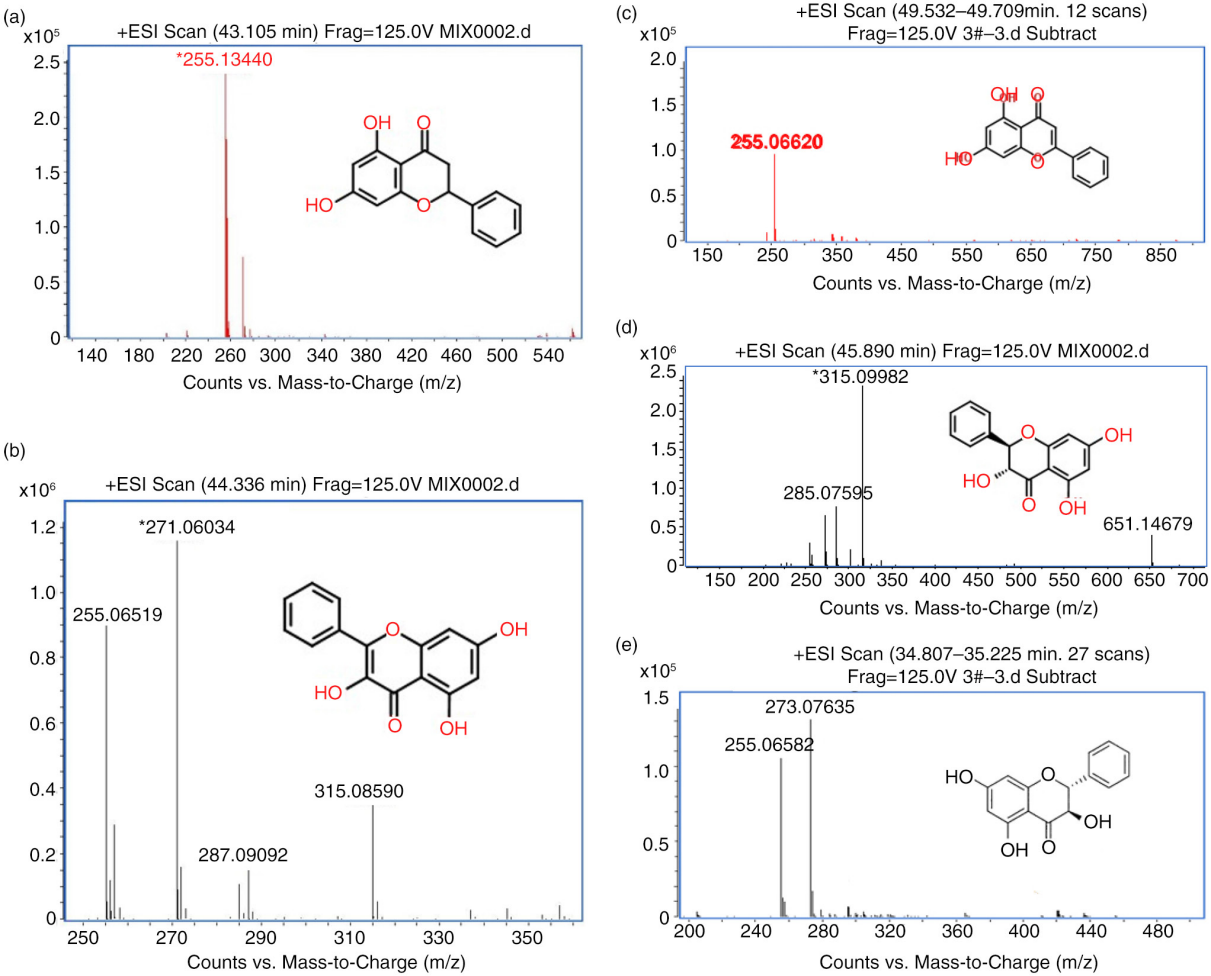
Spectrums of mass spectrometry for five compounds identification after HPLC purification from fraction three extracted from dichloromethane extracts of propolis. a) Pinocembrin in fraction 1; b) galangin in fraction 2; c) chrysin in fraction 3; d) pinobanksin 3-O-acetate in fraction 4; and e) pinobanksin in fraction 5.

**Fig. 4:**
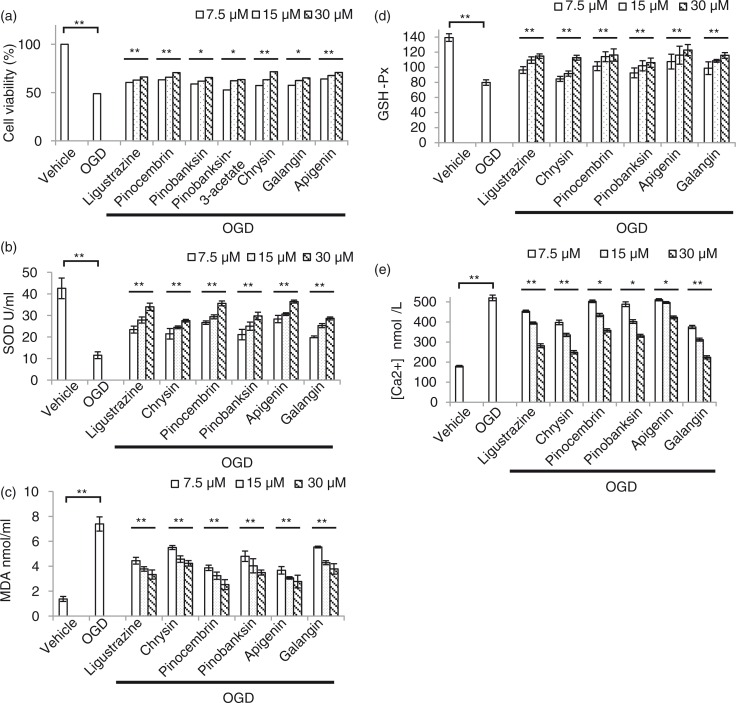
The cell-protection effects of five compounds from serial purification on an OGD-treated N2a cell. a) Cytotoxicity assay for all compounds in different dose. b) Compounds extracted from propolis enhanced superoxide dismutase (SOD) to protect cell from damage induced by OGD. c) Down-regulated oxidative stress marker, malondialdehyde (MDA), in propolis extracts treated N2a cell after OGD induction. d) The antioxidant, glutathione (GSH), was increased in propolis extracts treated N2a cell after OGD induction. e) Ca^2+^ influx was reduced in propolis extracts treated N2a cell after OGD induction. *n*=3 for each group; *p<0.05; **p<0.01; compound-treated groups were compared with the OGD-treated only group.

Five compounds from Fr.3 were further separated by HPLC and followed by MS identification, including pinocembrin (Fig. 3a), galangin (Fig. 3b), chrysin (Fig. 3c), pinobanksin 3-O-acetate (Fig. 3d), and pinobanksin (Fig. 3e). To test the cytotoxicity of these compounds, the dosage of IC50 was evaluated by MTT assay after a different dosage treatment (Fig. 4a). Among all compounds, cell viability was over 50%, while the compound treatment at concentration was as high as 30 µM. Therefore, 7.5 µM, 15 µM, and 30 µM were used for following dosage experiments. OGD can elevate oxidative stress, and superoxide dismutase (SOD) plays a protective role in ischemia after its activation. Thus, we evaluated the level of SOD after OGD induction with and without compound treatment (Fig. 4b). Chrysin, pinocembrin, pinobanksin, apigenin, and galangin can enhance SOD level in a dosage-dependent manner. On the other hand, malondialdehyde (MDA), an oxidative stress marker, was also reduced if treated with all compounds in a dosage-dependent manner (Fig. 4c). The antioxidant glutathione (GSH) also increased after treatment in an OGD-challenged condition (Fig. 4d). Since oxidative stress caused Ca^2+^ influx into the cytoplasm from the extracellular environment, we tested the Ca^2+^ in the cell after treatment (Fig. 4e). The results indicated Ca^2+^ was decreased after treatment in OGD stress. Taken together, these results suggested that purified compounds from propolis can attenuate cell damage due to OGD induction through anti-oxidation pathways.

### Purified compounds from propolis extracts can protect cell from OGD-induced apoptosis

**Fig. 5:**
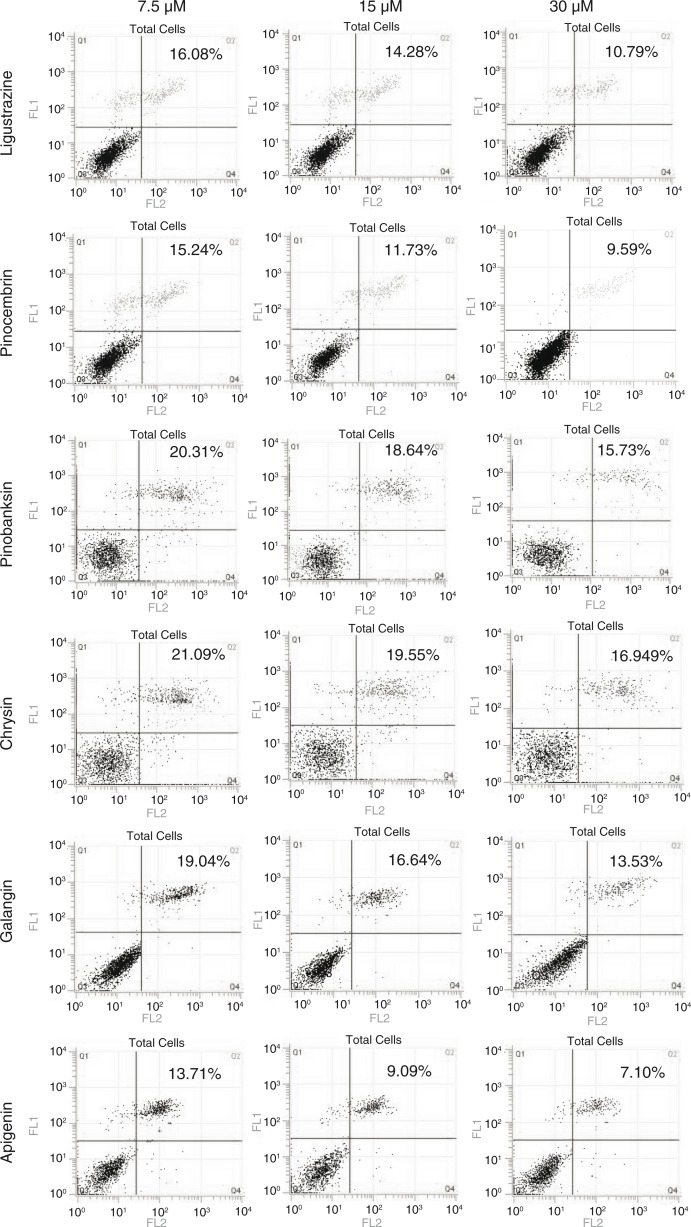
Compounds from propolis extracts can inhibit apoptosis induced by OGD in N2a cell. Cell apoptosis induced by OGD and rescued by compound treatment was checked by flow cytometry.

**Fig. 6:**
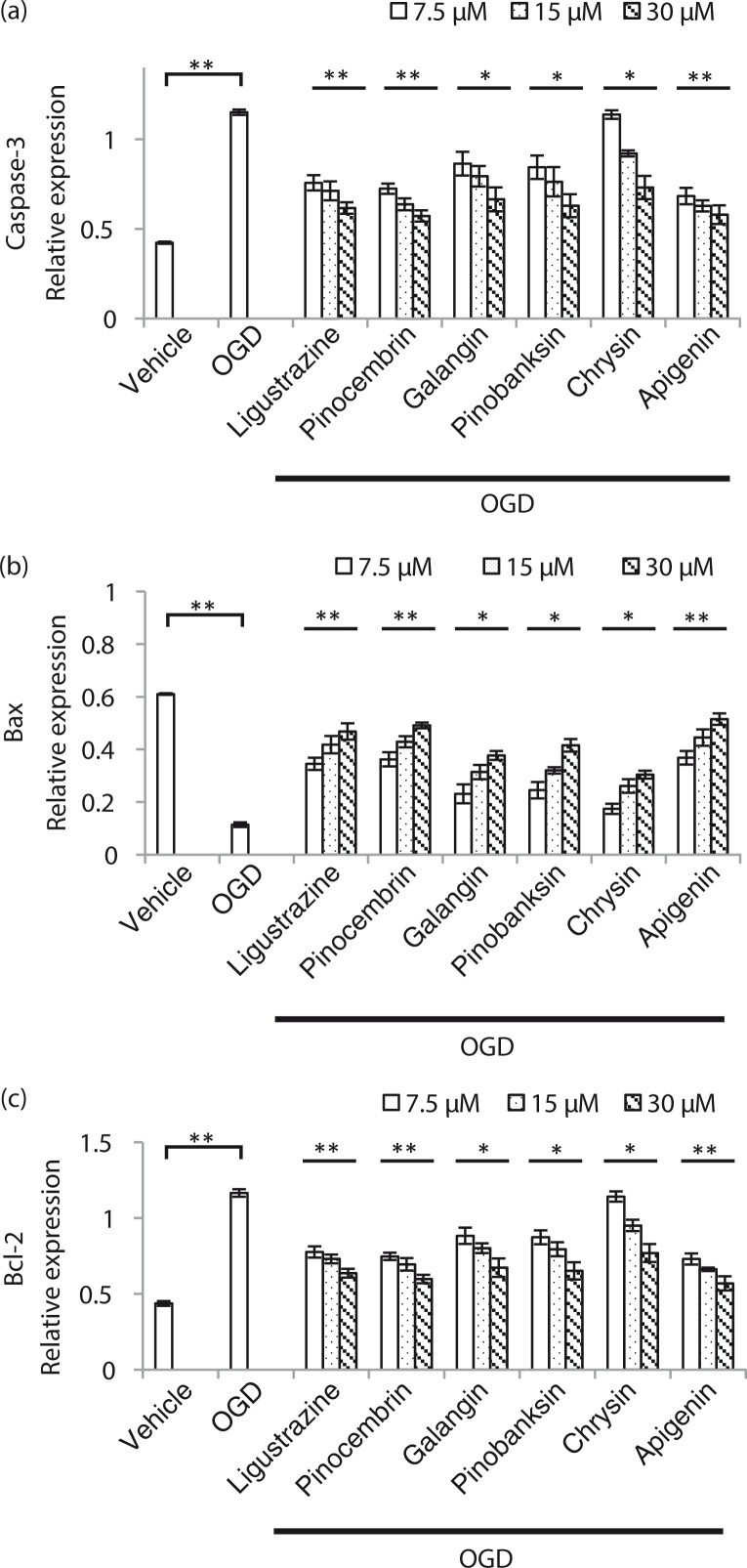
Apoptosis-related proteins were significantly altered in compounds from a propolis-treated OGD-induced N2a cell. a) Propolis extract compounds inhibited vaspase-3 expression level in N2a cell after OGD induction. b) Propolis extract compounds enhanced Bax expression level in an N2a cell after OGD induction. c) Propolis extract compounds inhibited Bcl-2 expression level in an N2a cell after OGD induction. n=3 for each group; *p<0.05; **p<0.01; compound-treated groups were compared with the OGD-treated only group.

In order to confirm the phenotype, cell apoptosis induced by OGD and rescued by compound treatment was checked by flow cytometry (Fig. 5). By increasing the concentration of five compounds, cell apoptosis induced by OGD was reduced. We also evaluated the apoptosis-related protein expression by Western blot followed by quantification (Fig. 6). Activated caspase-3, an apoptosis marker, was reduced (Fig. 6a); Bax, another marker for apoptosis, was elevated (Fig. 6b); and Bcl-2 also exhibited a decreased trend in a dosage-dependent manner (Fig. 6c). We concluded that compounds from propolis extracts can significantly prevent cell apoptosis induced by OGD.

## Discussion

Bee propolis was collected from tree buds and bark by bees, and mixed with a secretion of bee tongue gland and wax gland. It was processed by bees and transformed into a jelly substance by bees. Propolis is rich in terpenes, phenolics, flavonoids, and other bio-active substances, which are anticancer, antibacterial, anti-inflammatory, antioxidant, hepatoprotective, and protective of the brain. In recent years, propolis has become the focus of research in domestic and overseas research. In this paper, propolis was separated and studied for its effect on ischemia-reperfusion injury. In this study, OGD treated N2a cells, a model of cerebral ischemia reperfusion injury, was established and utilized for evaluating the effect of propolis extracts. Shandong propolis was separated into an ethanol extraction and organic solvent extraction, and further purified by column chromatography. HPLC were used to separating the bio-active substances of propolis anti-ischemia-reperfusion injury. Cell viability was measured by CCK-8 assay, and the results showed that the 70% ethanol extract group showed the highest protective effect. Column chromatography used to separate dichloromethane extracts and Fr.3 showed the highest protective effect. Furthermore, HPLC were used to separate Fr.3, and the five components, including pinocembrin, pinobanksin, pinobanksin-3-acetate, chrysin, and galangin, were finally purified. All of test extracts were used to evaluate the protective effect in OGD-treated N2a cells. The results showed that all five monomers showed the higher protective effect, especially apigenin and pinocembrin. Pinobanksin, galangin, and chrysin showed highly significant differences compared with the model group in middle and high concentrations.

Although I/R injury is a complex and systemic event that may involve a number of physiological processes, several processes, including free radical production, nitric oxide depletion, endothelial dysfunction, as well as cytokines production, were consensus issues in the injury process (28). Many therapeutic strategies were investigated to reduce injury during I/R damage, especially the physiological and pharmacological free radical scavengers like L-carnitine (29). One of the mechanisms by which propolis can protect cells from free radical damage due to I/R may be its anti-oxidative enhancement, which elevates anti-oxidant protein expression. The anti-oxidation of propolis experiment results supported this hypothesis that, after pinocembrin, chrysin, pinobanksin, galangin, and apigenin treatment, the MDA content in cells decreased significantly while the SOD and GSH-Px activity was enhanced in cells, thereby reducing ischemia and the reperfusion-injury-induced oxidative damage to cells. Pinocembrin and galangin showed a higher antioxidant effect than ligustrazine. Another mechanism of cerebral ischemia-reperfusion injury was cell damage caused by calcium overload. In the previous study, accumulations of intracellular calcium may promote neural death in many pathological disease processes, such as cerebral ischemia or epilepsy (30, 31). The intracellular free calcium concentration determination experiment showed that, after treatment by the five compounds of propolis, the intracellular free calcium concentration of N2a cells decreased significantly, suggesting that inhibiting intracellular calcium overload can reduce the severity of N2a cell injury, thereby antagonizing the ischemia reperfusion cell injury caused by OGD. Interestingly, Schild et al. indicated that the mitochondrial membrane highly controls the ROS-related oxidative stress that is involved in the permeability of brain mitochondria and calcium concentration during hypoxia/reoxygenation in the brain (32). In another study, the role of the mitochondrial membrane potential in ROS generation during I/R was a focus (33). Whether propolis eliminated the oxidative stress via modulating mitochondrial membrane is a possibility that needs further investigation. For I/R-injury-induced apoptosis, the anti-apoptosis of propolis experiment results showed that the five compounds of propolis can effectively reduce the apoptosis of N2a cells induced by OGD by up-regulating the expression of Bcl-2 mRNA and down-regulating caspase-3 as well as Bax expression. Taken together, propolis can protect N2a cell from OGD-induced damage via decreasing calcium concentration or inhibiting the apoptosis pathway.

This study is focused on the bio-active substances of propolis in anti-ischemia-reperfusion injury, anti-oxidation, modulating intracellular free calcium concentration, and anti-apoptosis on the cerebral I/R injury cell model. Of all the extracts, pinocembrin and galangin are the two compounds with highest bio-activities of propolis, even higher than those of ligustrazine. This is the first time that the extracts of propolis have been studied in the field of anti-oxidation. To develop related products from propolis extracts has a potential benefit for the treatment in patients with cardiovascular and cerebrovascular diseases, and even for prevention in people with related risks.

In conclusion, this study mentioned the separation and purification of propolis and extracts, especially the dichloromethane parts in anti-oxidative stress. We demonstrated that it can be used against cerebral ischemia-reperfusion injury in the N2a cell model. Although the detail molecular mechanism needs further investigations and exploration, this is the first time the bioactivity of propolis on anti-ischemia-induced damage has been highlighted. The utility of propolis as a daily food supplement or in adjuvant therapeutic use should be given attention.
